# Supplementary material for: TENT5-mediated polyadenylation of mRNAs encoding secreted proteins is essential for gametogenesis in mice
Source: Nat Commun. 2024 Jun 22;15:5331. doi: 10.1038/s41467-024-49479-4 (PMC11193744; doi:10.1038/s41467-024-49479-4)

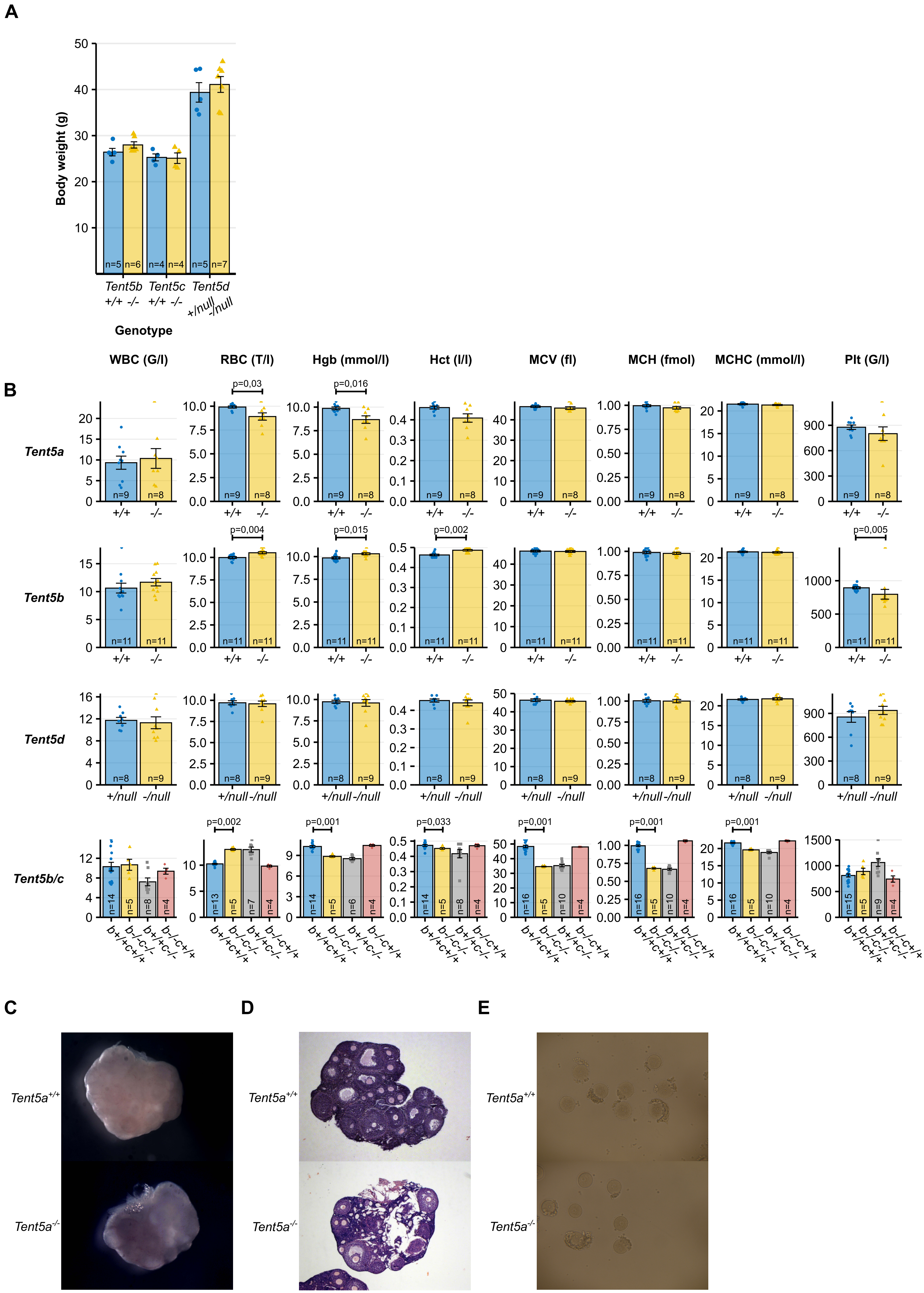
A
Body weight (g)
Genotype
Tent5b
Tent5c
Tent5d
B
WBC (G/l)
RBC (T/l)
Hgb (mmol/l)
Hct (l/l)
MCV (fl)
MCH (fmol)
MCHC (mmol/l)
Plt (G/l)
Tent5a
Tent5b
Tent5d
Tent5b/c
C
D
E
Tent5a+/+
Tent5a-/-

Supplementary Figure 1. Basic phenotype analysis of all *Tent5* KO mice lines.

A. Body weight of males of different *Tent5* KO mice lines. Animals of each mouse line were weighted at different age. Individual data points and n values represent individual animals weighted, bars represent mean values, error bars represent SEM, p-values reported for comparison in the Mann-Whitney-Wilcoxon test. **B.** Blood morphology parameters related to the *Tent5* mice lines genotype. Multiple differences in *Tent5b*$^{-/-}$ *Tent5c*$^{-/-}$ females are related to a detrimental effect of *Tent5c*$^{-/-}$ mutation alone, reported previously by Mroczek *et al.*[27]. Individual data points and n values represent blood samples from individual animals, bars represent mean values, error bars represent SEM; p-values reported for mean parameter value comparison in Mann-Whitney-Wilcoxon test; WBC = white blood cells, RBC = red blood cells, Hgb = hemoglobin, Hct = hematocrit, MCV = mean corpuscular volume, MCH = mean cell hemoglobin, MCHC = mean corpuscular hemoglobin concentration, Plt = Platelet. **C-E.** *Tent5a*$^{-/-}$ females' ovary morphology (**C**), histology (**D**) and GV oocytes (**E**) compared to *Tent5a*$^{+/+}$ ones. No changes observed in follicle growth, ovulation, and oocyte condition at GV stage.

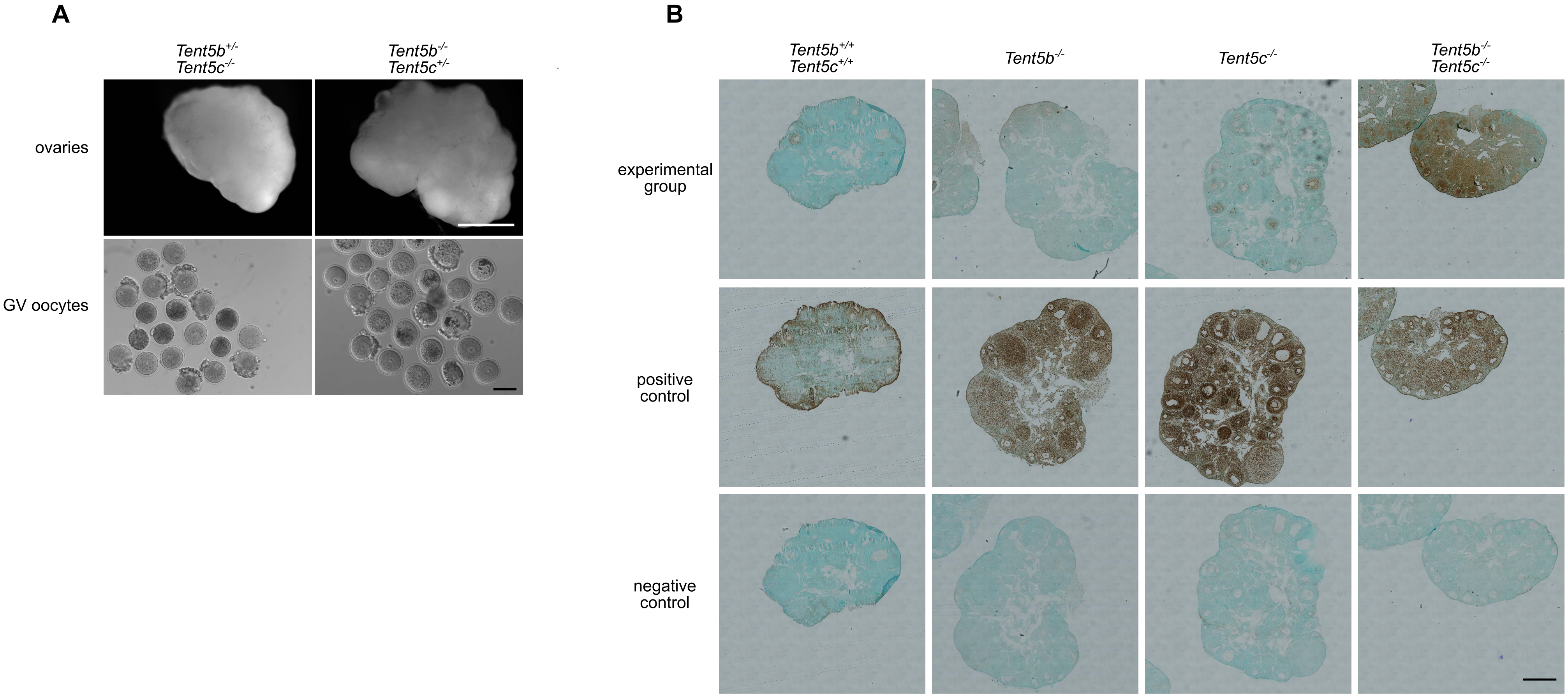
A
Tent5b+/-
Tent5c-/-
Tent5b-/-
Tent5c+/-
ovaries
GV oocytes
B
Tent5b+/+
Tent5c+/+
Tent5b-/-
Tent5c-/-
Tent5b-/-
Tent5c-/-
experimental group
positive control
negative control

Supplementary Figure 2. *Tent5b/c* dKO ovaries and oocytes morphology and apoptosis analysis

A. Morphology of the ovaries and GV oocytes of females with one wild-type allele of either *Tent5b* or *Tent5c* gene, which is sufficient to maintain normal fertility. **B**. TUNEL assay for signs of apoptosis in cross-section of ovaries of different *Tent5b* and *Tent5c* genotypes. Only *Tent5b*$^{-/-}$ *Tent5c*$^{-/-}$ mice display high levels of apoptosis in both oocytes and somatic granulosa cells surrounding them.

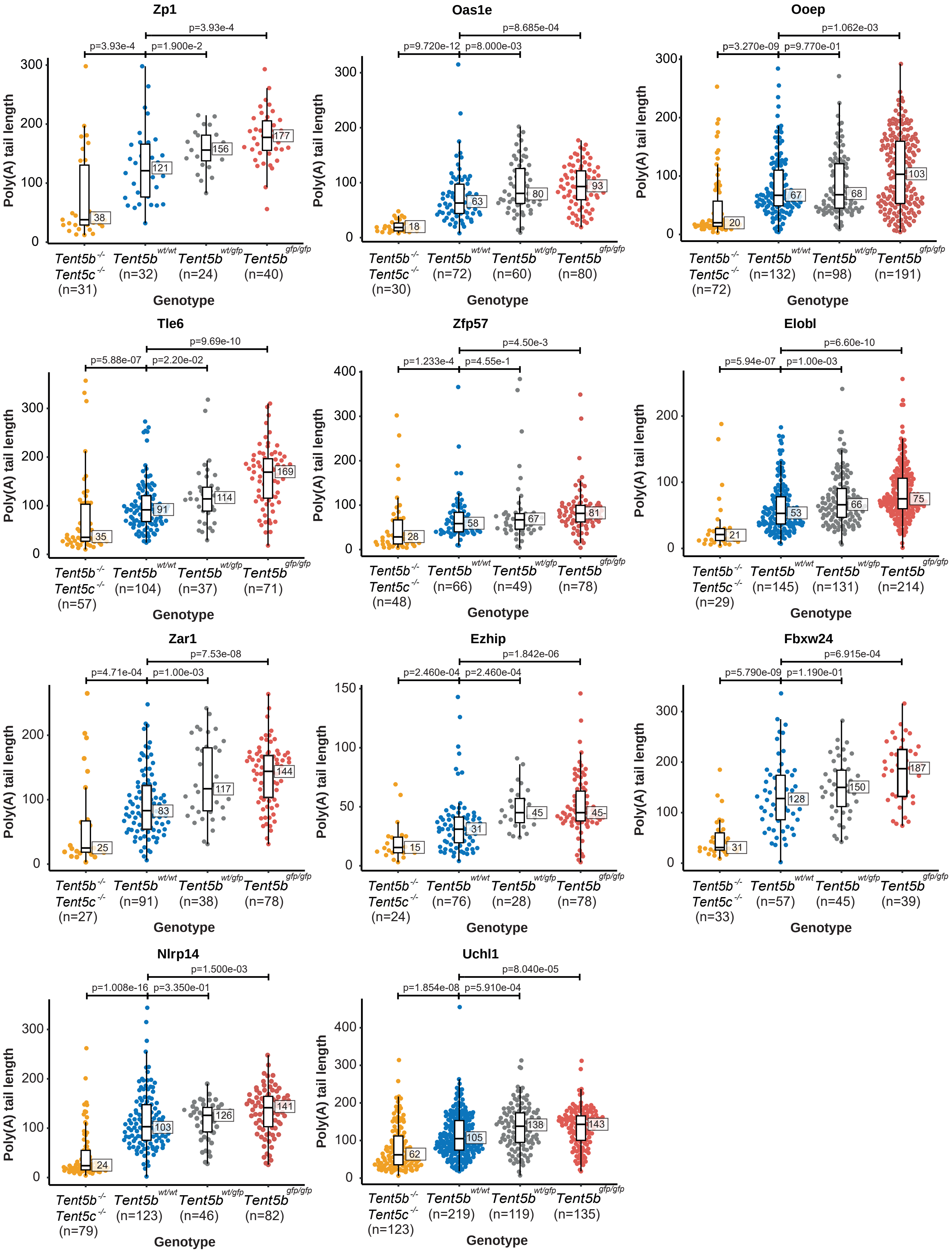
Zp1
p=3.93e-4
p=3.93e-4
p=1.900e-2
38
121
156
177
Oas1e
p=8.685e-04
p=9.720e-12
p=8.000e-03
18
63
80
93
Ooep
p=1.062e-03
p=3.270e-09
p=9.770e-01
20
67
68
103
Tle6
p=9.69e-10
p=5.88e-07
p=2.20e-02
35
91
114
169
Zfp57
p=4.50e-3
p=1.233e-4
p=4.55e-1
28
58
67
81
Elobl
p=6.60e-10
p=5.94e-07
p=1.00e-03
21
53
66
75
Zar1
p=7.53e-08
p=4.71e-04
p=1.00e-03
25
83
117
144
Ezhip
p=1.842e-06
p=2.460e-04
p=2.460e-04
15
31
45
45
Fbxw24
p=6.915e-04
p=5.790e-09
p=1.190e-01
31
128
150
187
Nlrp14
p=1.500e-03
p=1.008e-16
p=3.350e-01
24
103
126
141
Uchl1
p=8.040e-05
p=1.854e-08
p=5.910e-04
62
105
138
143
Poly(A) tail length
Genotype
Tent5b-/- Tent5c-/-
Tent5b wt/wt
Tent5b wt/gfp
Tent5b gfp/gfp
Zp1: (n=31) (n=32) (n=24) (n=40)
Oas1e: (n=30) (n=72) (n=60) (n=80)
Ooep: (n=72) (n=132) (n=98) (n=191)
Tle6: (n=57) (n=104) (n=37) (n=71)
Zfp57: (n=48) (n=66) (n=49) (n=78)
Elobl: (n=29) (n=145) (n=131) (n=214)
Zar1: (n=27) (n=91) (n=38) (n=78)
Ezhip: (n=24) (n=76) (n=28) (n=78)
Fbxw24: (n=33) (n=57) (n=45) (n=39)
Nlrp14: (n=79) (n=123) (n=46) (n=82)
Uchl1: (n=123) (n=219) (n=119) (n=135)

Supplementary Figure 3. TENT5s polyadenylate mRNAs in oocytes, which tight regulation is essential for oogenesis.

DRS-based poly(A) tail lengths profiling of mRNAs isolated from ovaries. Median poly(A) tail lengths are plotted in white rectangles, p-values reported for comparison in Mann-Whitney-Wilcoxon test with Bonferroni Hallberg correction.

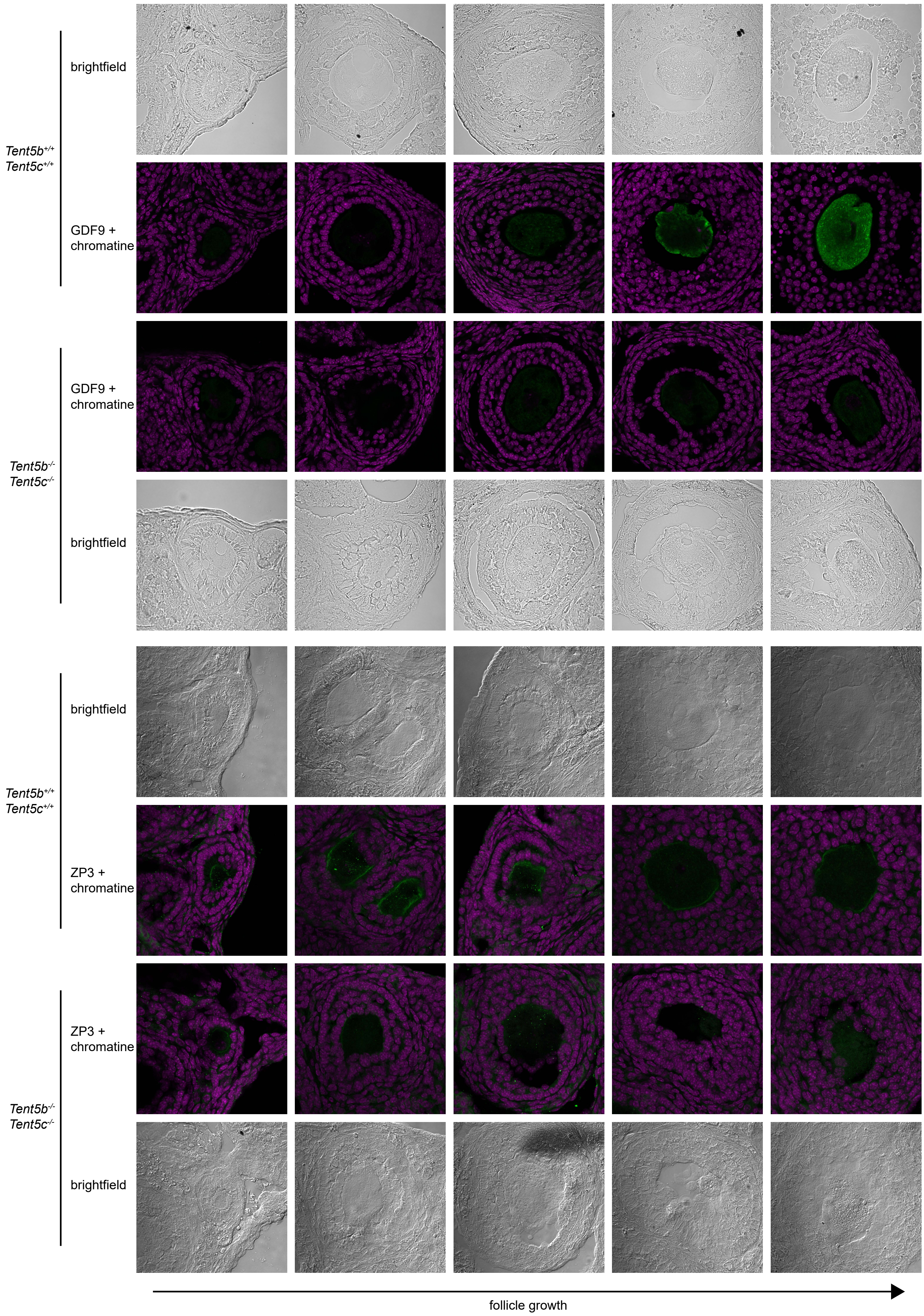
brightfield
Tent5b+/+
Tent5c+/+
GDF9 +
chromatine
GDF9 +
chromatine
Tent5b-/-
Tent5c-/-
brightfield
brightfield
Tent5b+/+
Tent5c+/+
ZP3 +
chromatine
ZP3 +
chromatine
Tent5b-/-
Tent5c-/-
brightfield
follicle growth

Supplementary Figure 4. Ovary immunohistochemistry staining

Immunohistochemistry staining of GDF9 (green), ZP3 (green) and chromatin (magenta) in ovaries of *Tent5b*$^{-/-}$ *Tent5c*$^{-/-}$ and *Tent5b*$^{+/+}$ *Tent5c*$^{+/+}$ females. Scale bar = 50 µm.

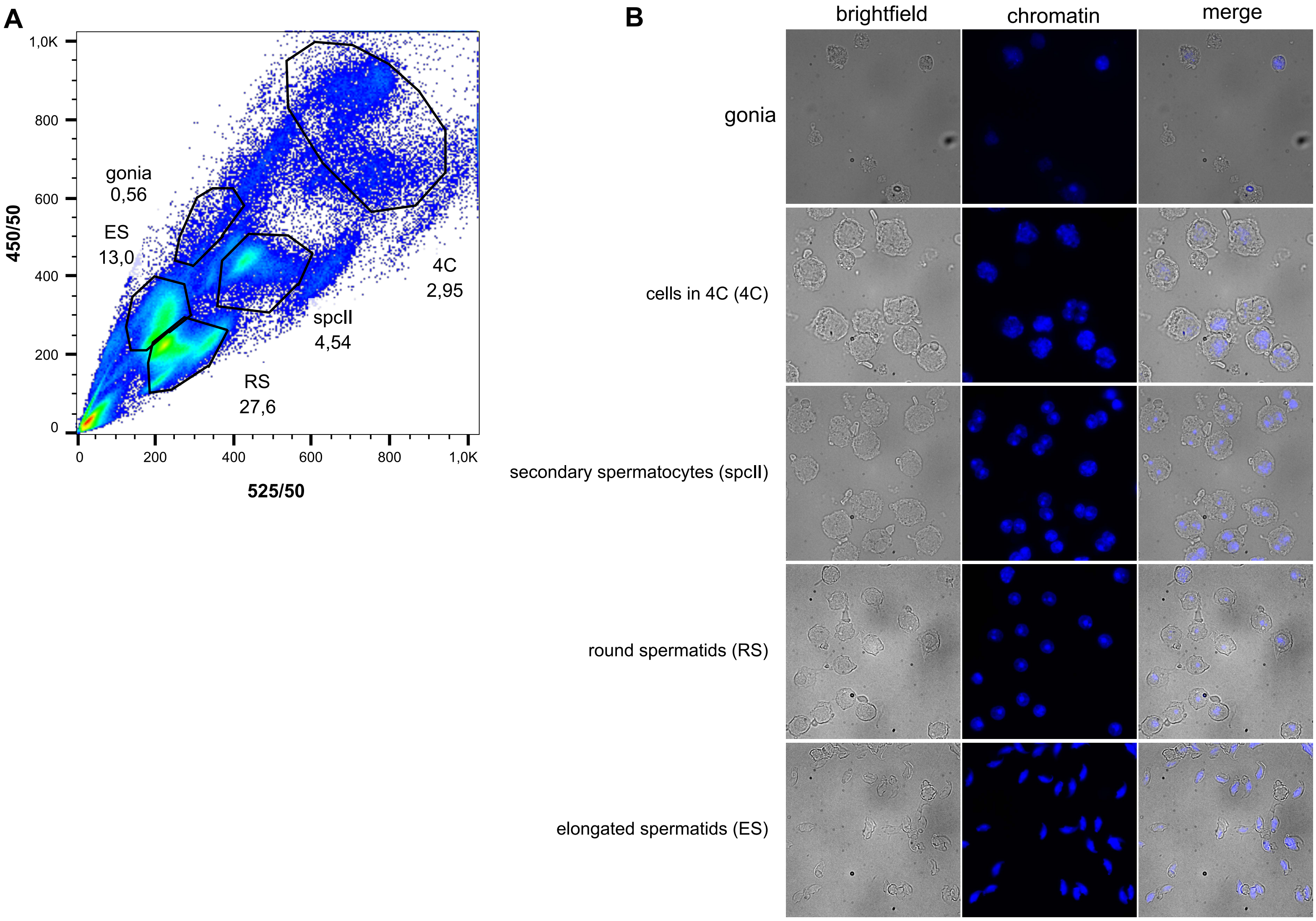
A
B
gonia
0,56
ES
13,0
4C
2,95
spcII
4,54
RS
27,6
450/50
525/50
brightfield
chromatin
merge
gonia
cells in 4C (4C)
secondary spermatocytes (spcII)
round spermatids (RS)
elongated spermatids (ES)

Supplementary Figure 5. Male germ cells sorting

A. Gating example of cells on different stages of spermatogenesis based on cells' size, shape, and DNA content in FACS analysis. **B**. Morphology and DNA staining pictures confirming cell sorting procedures correctness. Cells at different spermatogenesis stages display different patterns of DNA staining, size and shape.

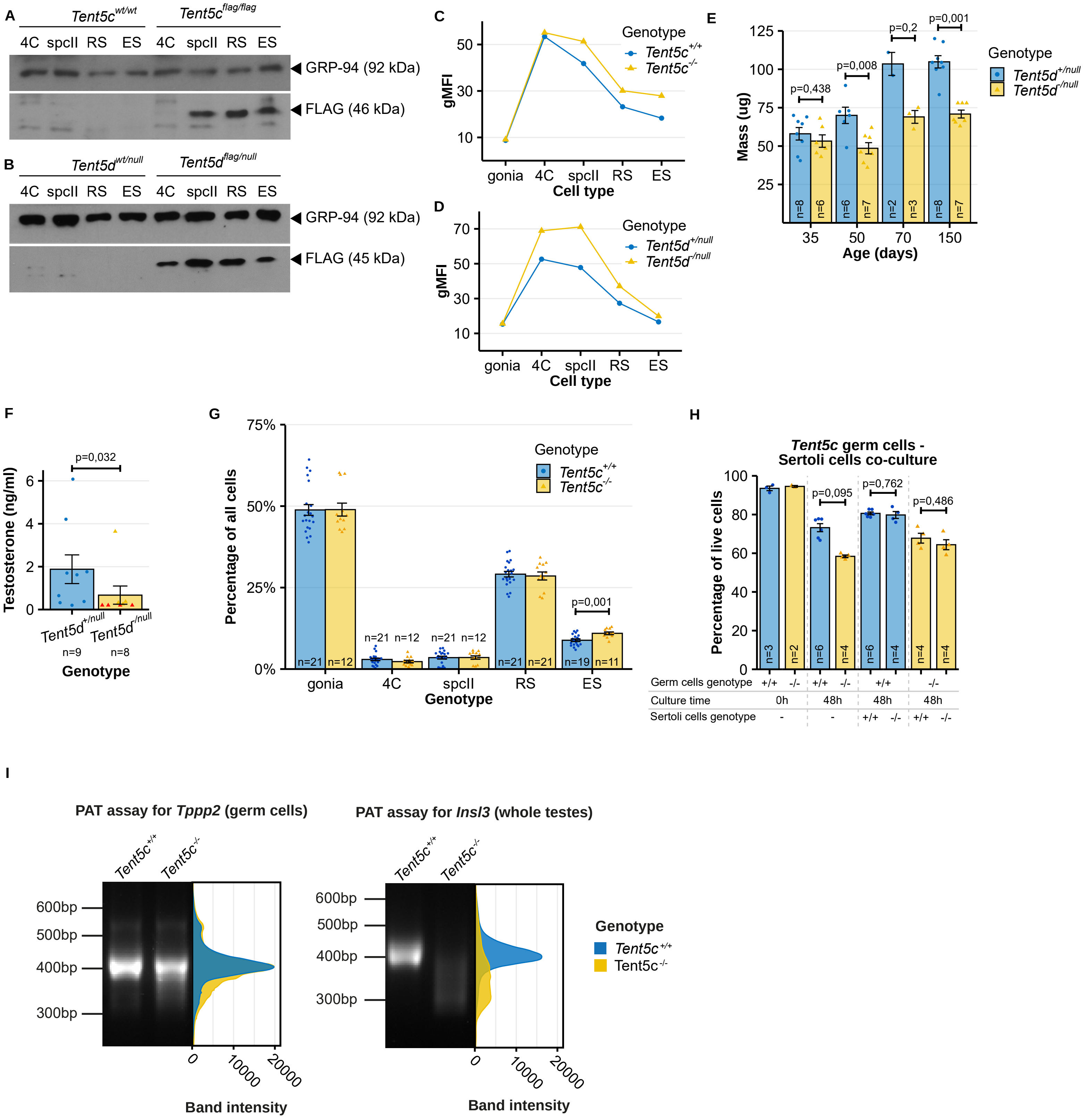
A
Tent5c wt/wt
Tent5c flag/flag
4C spcII RS ES 4C spcII RS ES
GRP-94 (92 kDa)
FLAG (46 kDa)
B
Tent5d wt/null
Tent5d flag/null
4C spcII RS ES 4C spcII RS ES
GRP-94 (92 kDa)
FLAG (45 kDa)
C
gMFI
Cell type
gonia 4C spcII RS ES
Genotype
Tent5c +/+
Tent5c -/-
D
gMFI
Cell type
gonia 4C spcII RS ES
Genotype
Tent5d +/null
Tent5d -/null
E
Mass (ug)
Age (days)
35 50 70 150
p=0,438
p=0,008
p=0,2
p=0,001
n=8 n=6 n=6 n=7 n=2 n=3 n=8 n=7
Genotype
Tent5d +/null
Tent5d -/null
F
Testosterone (ng/ml)
p=0,032
Tent5d +/null
Tent5d -/null
n=9
n=8
Genotype
G
Percentage of all cells
75% 50% 25% 0%
Genotype
Tent5c +/+
Tent5c -/-
n=21 n=12 n=21 n=12 n=21 n=12 n=21 n=21 n=19 n=11
p=0,001
gonia 4C spcII RS ES
Genotype
H
Tent5c germ cells -
Sertoli cells co-culture
Percentage of live cells
p=0,095
p=0,762
p=0,486
n=3 n=2 n=6 n=4 n=6 n=4 n=4 n=4
Germ cells genotype +/+ -/- +/+ -/- +/+ -/-
Culture time 0h 48h 48h 48h
Sertoli cells genotype - - +/+ -/- +/+ -/-
I
PAT assay for Tppp2 (germ cells)
PAT assay for Insl3 (whole testes)
Tent5c +/+
Tent5c -/-
600bp
500bp
400bp
300bp
0 10000 20000
Band intensity
Genotype
Tent5c +/+
Tent5c -/-

Supplementary Figure 6. Analysis of *Tent5c* and *Tent5d* KO phenotype in males

A-B. Western blot analysis of FLAG-tagged TENT5C and TENT5D expression in different stages of spermatogenesis. Antibodies against GRP-94 were used as a loading control. Black arrowheads mark position of detected proteins. This experiment had one technical repetition. **C-D.** Cytometric analysis of GFP-tagged TENT5C and TENT5D expression in different stages of spermatogenesis, presented as changes as gMFI (geometric mean fluorescence intensity). **E.** Testes mass changes in *Tent5d*$^{+/null}$ and *Tent5d*$^{-/null}$ males in life. Individual data points and n values represent testes from individual males weighted, bars represent mean values, error bars represent SEM, p-values reported for comparison in Mann-Whitney-Wilcoxon test. **F.** Blood testosterone level in adult *Tent5d*$^{+/null}$ and *Tent5d*$^{-/null}$ males. Individual data points and n values represent samples from individual males, bars represent mean values, error bars represent SEM, p-values reported for comparison in Mann-Whitney-Wilcoxon test. **G.** Percentage of germ cells at different spermatogenesis stages among all isolated germ cells in *Tent5c*$^{+/+}$ and *Tent5c*$^{-/-}$ males. Individual data points and n values represent individual males from which germ cells were isolated, bars represent mean values, error bars represent SEM, p-value reported for comparison in t-test, two-tailed. **H.** Germ cell survival in 48h of *in vitro* culture depending on *Tent5c* genotype of germ cells and presence and genotype of Sertoli cells. Individual data points and n values represent cell culture of germ cells isolated from single male, bars represent mean values, error bars represent SEM; p-values reported for comparison in Mann-Whitney-Wilcoxon test. **I.** PAT assay visualizing poly(A) tail length distribution of *Tppp2* and *Insl3* transcripts PCR-amplified from whole mRNA isolated from germ cells (*Tppp2*) and whole testes (*Insl3*).

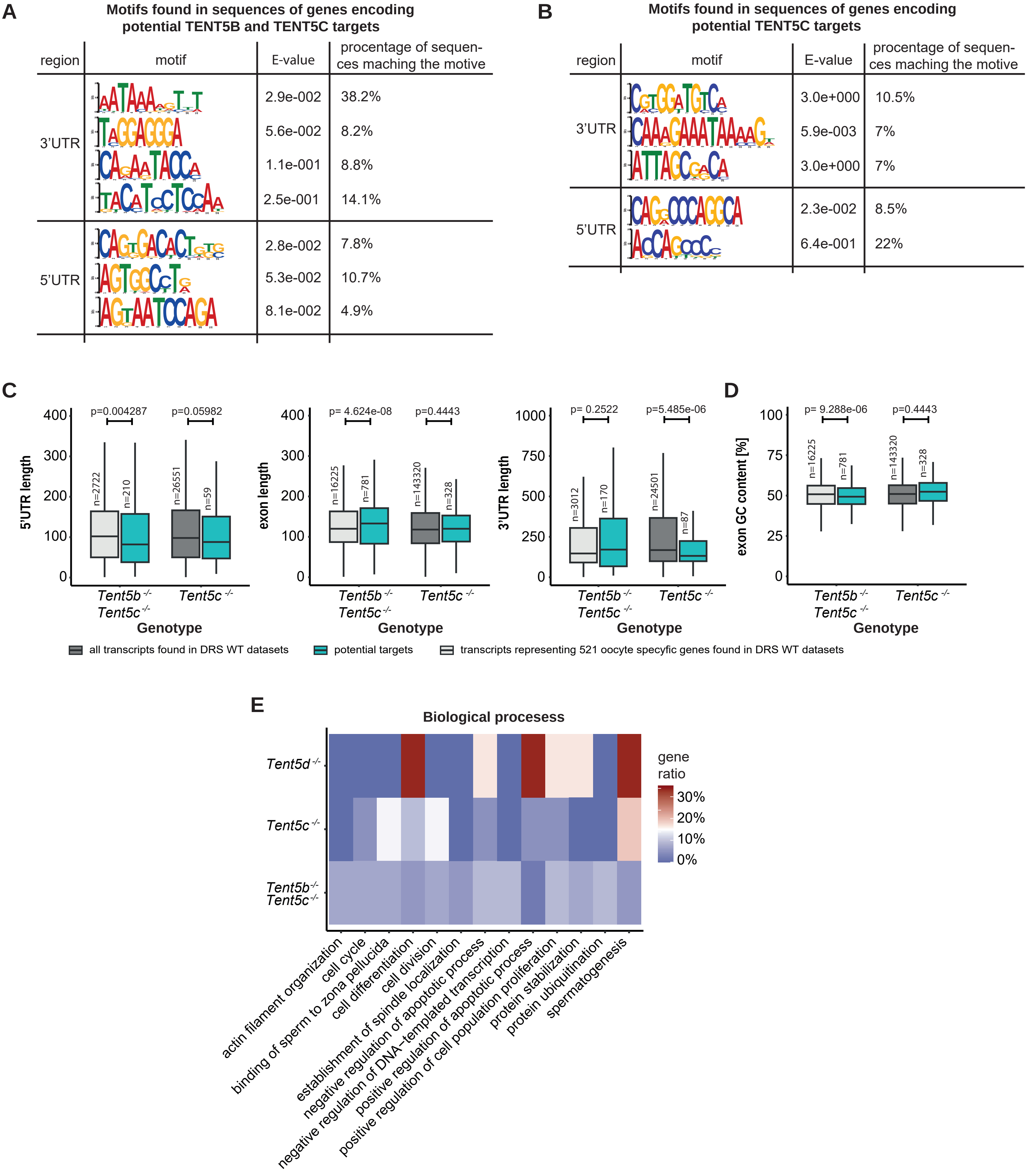
A
Motifs found in sequences of genes encoding potential TENT5B and TENT5C targets
region | motif | E-value | procentage of sequences maching the motive
3'UTR
2.9e-002 | 38.2%
5.6e-002 | 8.2%
1.1e-001 | 8.8%
2.5e-001 | 14.1%
5'UTR
2.8e-002 | 7.8%
5.3e-002 | 10.7%
8.1e-002 | 4.9%
B
Motifs found in sequences of genes encoding potential TENT5C targets
region | motif | E-value | procentage of sequences maching the motive
3'UTR
3.0e+000 | 10.5%
5.9e-003 | 7%
3.0e+000 | 7%
5'UTR
2.3e-002 | 8.5%
6.4e-001 | 22%
C
5'UTR length
p=0.004287
p=0.05982
n=2722
n=210
n=26551
n=59
exon length
p= 4.624e-08
p=0.4443
n=16225
n=781
n=143320
n=328
3'UTR length
p= 0.2522
p=5.485e-06
n=3012
n=170
n=24501
n=87
D
exon GC content [%]
p= 9.288e-06
p=0.4443
n=16225
n=781
n=143320
n=328
Tent5b -/- Tent5c -/-
Tent5c -/-
Genotype
all transcripts found in DRS WT datasets
potential targets
transcripts representing 521 oocyte specyfic genes found in DRS WT datasets
E
Biological processes
Tent5d -/-
Tent5c -/-
Tent5b -/- Tent5c -/-
gene ratio
30%
20%
10%
0%
actin filament organization
binding of sperm to zona pellucida
cell cycle
cell differentiation
cell division
establishment of spindle localization
negative regulation of apoptotic process
negative regulation of DNA-templated transcription
positive regulation of apoptotic process
positive regulation of cell population proliferation
protein stabilization
protein ubiquitination
spermatogenesis

Supplementary Figure 7. Motif and Gene ontology analysis of DRS results

A-B. 3`UTR, 5’UTR motif analysis did not reveal any enriched motifs. In CDS only in Tent5B/C substrates, short A-rich motive is significantly enriched. **C.** Differences in 5’UTR, 3’UTR and exon length between potential targets of TENT5 proteins compared to the rest of the transcriptome detected by DRS. In the ovaries, analyses were performed for 522 oocyte-enriched mRNAs. Mann-Whitney-Wilcoxon rank sum test was calculated. **D.** Difference in GC content between potential targets of Tent5 proteins. Mann-Whitney-Wilcoxon rank sum test was calculated. **E.** Gene ontology (GO) analysis of potential TENT5 protein targets.

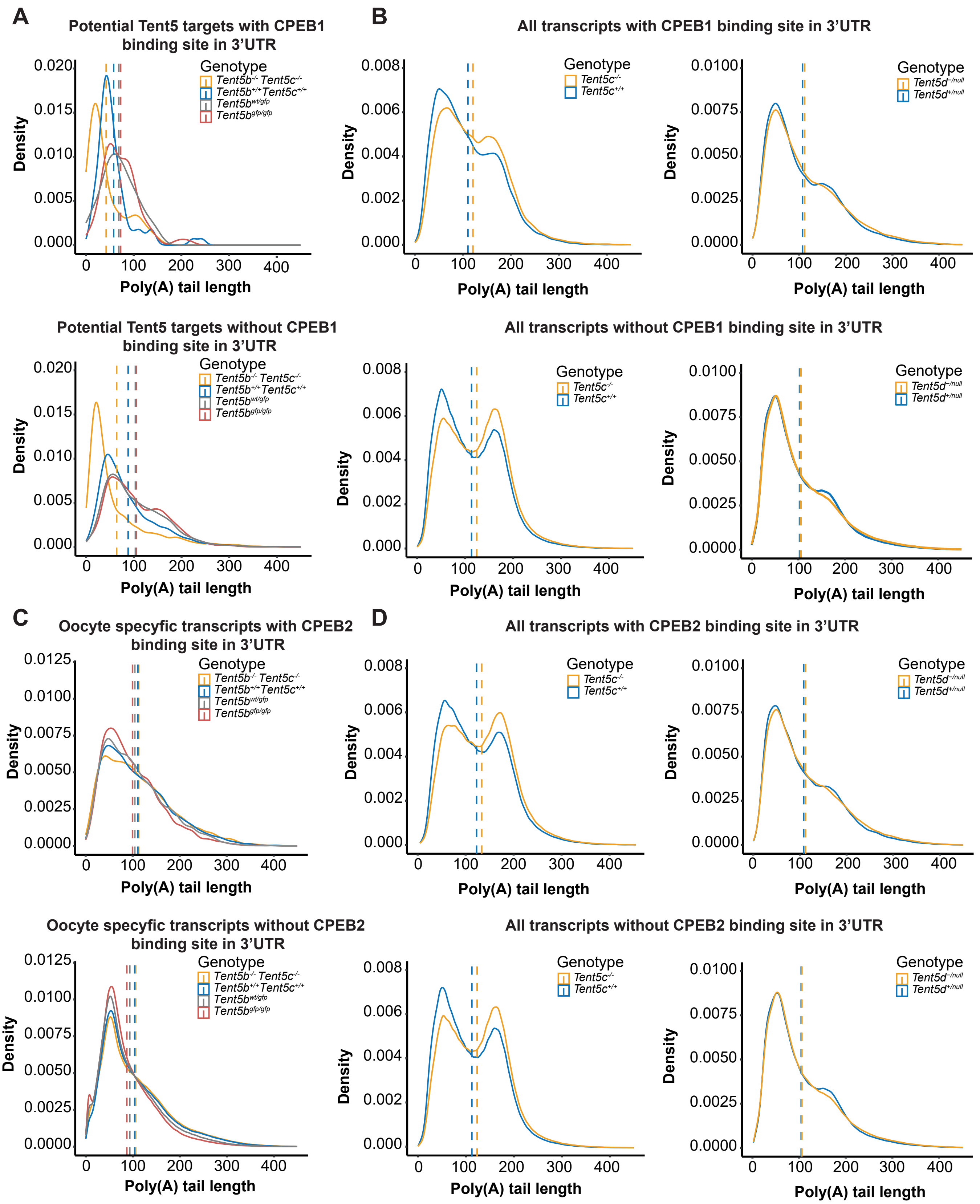

A
Potential Tent5 targets with CPEB1 binding site in 3’UTR
Genotype
Tent5b-/- Tent5c-/-
Tent5b+/+ Tent5c+/+
Tent5bwt/gfp
Tent5bgfp/gfp
Density
Poly(A) tail length
B
All transcripts with CPEB1 binding site in 3’UTR
Genotype
Tent5c-/-
Tent5c+/+
Tent5d-/null
Tent5d+/null
Density
Poly(A) tail length
Potential Tent5 targets without CPEB1 binding site in 3’UTR
All transcripts without CPEB1 binding site in 3’UTR
C
Oocyte specyfic transcripts with CPEB2 binding site in 3’UTR
D
All transcripts with CPEB2 binding site in 3’UTR
Oocyte specyfic transcripts without CPEB2 binding site in 3’UTR
All transcripts without CPEB2 binding site in 3’UTR

Supplementary Figure 8. CPEB1 and CPEB2 motifs analysis of DRS data

A. Changes in the global distribution of poly(A) tail lengths for group of transcripts selected as potential Tent5B/C targets. Mann-Whitney-Wilcoxon rank sum test was calculated. Dashed lines indicate the mean poly(A) tails lengths: Tent5b -/- Tent5c -/- - 42, Tent5b +/+ - 58, Tent5b wt/gfp - 69, Tent5b gfp/gfp - 73 for transcripts with CPEB1 and Tent5b-/- Tent5c-/- - 64, Tent5b+/+ - 88, Tent5b wt/gfp - 103, Tent5b gfp/gfp- 105 for transcripts without CPEB1. **B.** The global distribution of polyA tails lengths of RNA isolated from Tent5c +/+, Tent5c -/-, Tent5d +/+, Tent5d -/- mice. Mann-Whitney-Wilcoxon rank sum test was calculated. Dashed lines indicate the mean poly(A) tails lengths: Tent5c -/- - 130, Tent5c +/+ - 119, Tent5d -/- - 107, Tent5d +/+ - 112 for transcripts with CPEB1 and Tent5c -/- - 124, Tent5c +/+ - 113, Tent5d -/- - 105 , Tent5d +/+ - 103 for transcripts without CPEB1. **C.** The global distribution of polyA tails lengths of RNA isolated from Tent5b -/- Tent5c -/-, Tent5b +/+, Tent5b wt/gfp and Tent5b gfp/gfp mice. Mann-Whitney-Wilcoxon rank sum test was calculated. Dashed lines indicate the mean poly(A) tails lengths: Tent5b -/- Tent5c -/- - 116, Tent5b +/+ - 111, Tent5b wt/gfp - 107, Tent5b gfp/gfp - 99 for transcripts with CPEB2 and Tent5b-/- Tent5c-/- - 106, Tent5b+/+ - 104, Tent5b wt/gfp - 94, Tent5b gfp/gfp- 87 for transcripts without CPEB2. **D.** The global distribution of polyA tails lengths of RNA isolated from Tent5c +/+110, Tent5c -/- 127, Tent5d +/+, Tent5d -/- mice. Mann-Whitney-Wilcoxon rank sum test was calculated. Dashed lines indicate the mean poly(A) tails lengths: Tent5c -/- - 129, Tent5c +/+ - 118, Tent5d -/- - 112 , Tent5d +/+ - 108 for transcripts with CPEB2 and Tent5c -/- - 123, Tent5c +/+ - 112, Tent5d -/- - 104, Tent5d +/+ - 103 for transcripts without CPEB2.

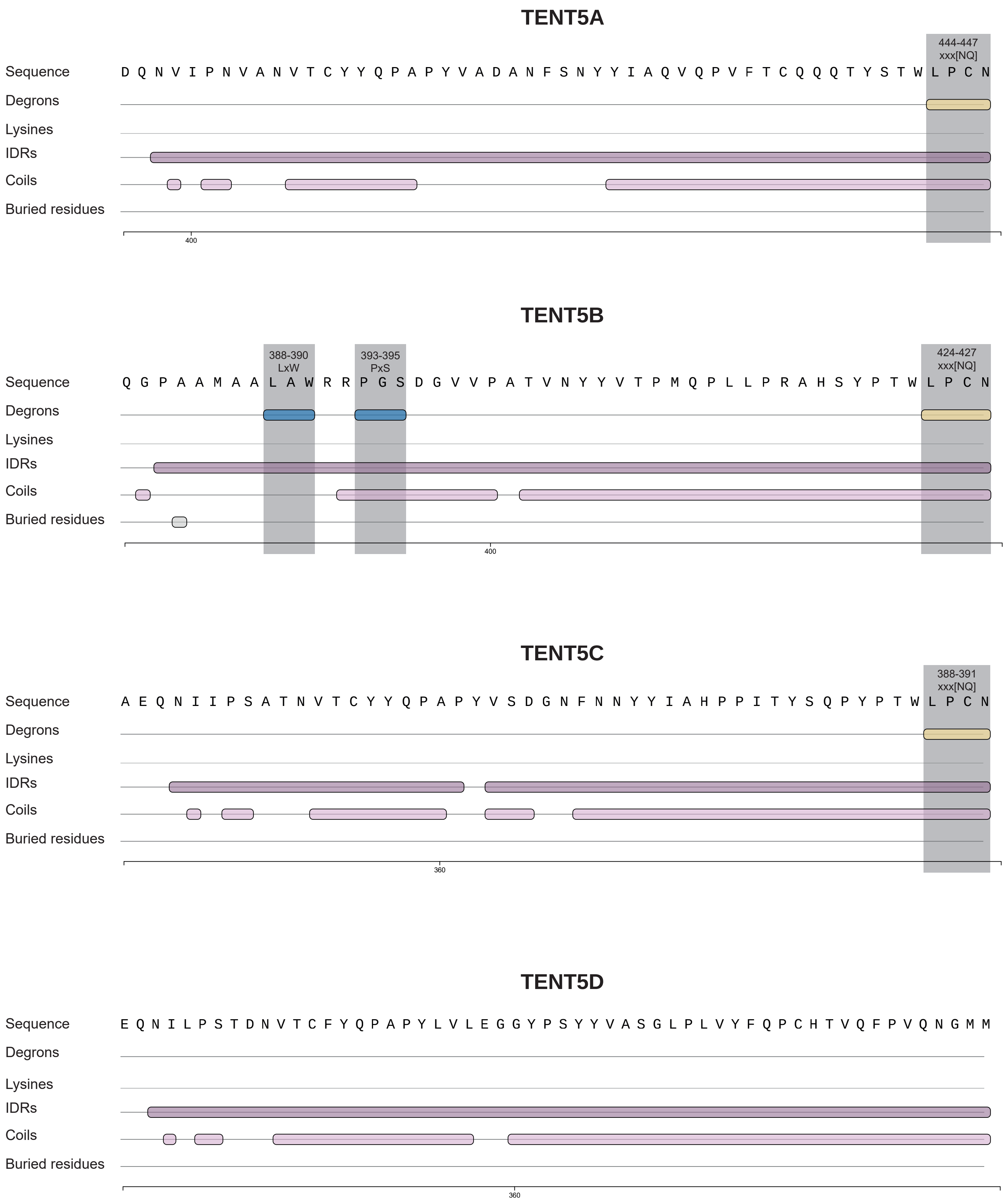
TENT5A
444-447
xxx[NQ]
Sequence
D Q N V I P N V A N V T C Y Y Q P A P Y V A D A N F S N Y Y I A Q V Q P V F T C Q Q Q T Y S T W L P C N
Degrons
Lysines
IDRs
Coils
Buried residues
400
TENT5B
388-390
LxW
393-395
PxS
424-427
xxx[NQ]
Sequence
Q G P A A M A A L A W R R P G S D G V V P A T V N Y Y V T P M Q P L L P R A H S Y P T W L P C N
Degrons
Lysines
IDRs
Coils
Buried residues
400
TENT5C
388-391
xxx[NQ]
Sequence
A E Q N I I P S A T N V T C Y Y Q P A P Y V S D G N F N N Y Y I A H P P I T Y S Q P Y P T W L P C N
Degrons
Lysines
IDRs
Coils
Buried residues
360
TENT5D
Sequence
E Q N I L P S T D N V T C F Y Q P A P Y L V L E G G Y P S Y Y V A S G L P L V Y F Q P C H T V Q F P V Q N G M M
Degrons
Lysines
IDRs
Coils
Buried residues
360

Supplementary Figure 9. Degrons in C-terminus of TENT5 proteins

Degron motifs detected in C-terminal region of TENT5 proteins using DEGRONOPEDIA. While C-terminal xxx[NQ] degron within intrinsically disordered region (IDR) is shared between TENT5A, B and C sequences, only TENT5B possesses two additional degron motifs within the same IDR.

Supplementary Table 1. Oligonucleotides, sgRNA and DNA repair templates sequences

Fw primer for genotyping of Tent5a KO mouse line (#2001): CAAGCCTGATTGTGAAGGTG	Gewartowska et al.[29]
Rv primer for genotyping of *Tent5a* KO mouse line (#2002): AAGGAAGAGAAGGAAACGCA	Gewartowska et al.[29]
Fw primer for genotyping of *Tent5b* KO mouse line (large deletion) (#2007): TTAGCCTGAAGACGCTATGG	
Rv primer for genotyping of Tent5b KO mouse line (large deletion) (#2008): GCATGGGGGTCACATAGTAA	
Fw primer for genotyping of *Tent5b* KO mouse line (small deletion) (#2005): AATCGAGGCTTAGCGAGTTA	
Rv primer for genotyping of *Tent5b* KO mouse line (small deletion) (#2006)	
Fw primer for genotyping of *Tent5c* KO mouse line (#2012): AGGTCCTGACTGAGGTCGTG	Mroczek et al.[27]
Rv primer for genotyping of *Tent5c* KO mouse line (#2013): TTCCTCAAAATCCCCGTACA	Mroczek et al.[27]
Fw primer for genotyping of Tent5d KO mouse line (#2016): TCGGAGATCAGATTCAGCAAT	This paper
Rv primer for genotyping of Tent5d KO mouse line (#2017): TAGTTCTACGTTCTTCCCGTT	This paper
Fw primer for genotyping of *Tent5b*-GFP and *Tent5b*-FLAG mouse lines (#2310): GTGGTCAACGAGAGCACAGTG	This paper
Rv1 primer for genotyping of *Tent5b*-GFP and *Tent5b*-FLAG mouse lines (#2311): gaagatggtgcgctcctgg	This paper
Fw primer for genotyping of GFP-*Tent5b* mouse line (#2160): atcagaaactcctgagagcc	This paper
Rv1 primer for genotyping of GFP-*Tent5b* mouse line (#2180): GAATGGGGATCGGCTCTTTC	This paper
Rv2 primer for genotyping of GFP-*Tent5b* mouse line (#2326): gacacgctgaacttgtggc	This paper
Fw primer for genotyping of *Tent5c*-GFP and *Tent5c*-FLAG mouse lines (#2014): CTTCAGAACCACTTCTCGGA	Mroczek et al.[27]
Rv primer for genotyping of *Tent5c*-GFP and *Tent5c*-FLAG mouse lines (#2015): AGAAGTCACGCCTCCTATTG	Mroczek et al.[27]
Fw primer for genotyping of *Tent5d*-GFP and *Tent5d*-FLAG mouse lines (#2018): CCCAGTACAGACAACGTAAC	This paper
Rv primer for genotyping of *Tent5d*-GFP and *Tent5d*-FLAG mouse lines (#2019): GTGTTCTTTCATACGTTAGCC	This paper
gRNA used for generation of *Tent5b* KO mouse line: TGTAGCCTAGGCCGCTCTC	This paper
gRNA used for generation of *Tent5d* KO mouse line: ACATACTCGCAAGCCATAA	This paper
gRNA used for generation of *Tent5b*-GFP and *Tent5b*-FLAG mouse lines: AGGATCAGAGTCAGTTGCA	This paper
dsDNA used as repair template for generation of *Tent5b*-FLAG mouse line: ACCCCCATGCAGCCACTGCTGCCCCGAGCTCACTCCTATCCTACCTGGCTG CCTTGCAACGACTACAAAGACGATGACGACAAGTGACTCTGATCCTGGCCA GAAGGGAATGAGCGCCATGGGGTGGGGTGGGGTCATCAGGTA	This paper

dsDNA used as repair template for generation of *Tent5b*-GFP mouse line:<br>ACCCCCATGCAGCCACTGCTGCCCCGAGCTCACTCCTATCCTACCTGGCTG<br>CCTTGCAACgagaatttgtattttcagggtgatatcatggtgagcaagggcgaggagctgttcaccggggt<br>ggtgcccatcctggtcgagctggacggcgacgtaaacggccacaagttcagcgtgtccggcgagggcgagg<br>gcgatgccacctacggcaagctgaccctgaagttcatctgcaccaccggcaagctgcccgtgccctggccca<br>ccctcgtgaccaccctgacctacggcgtgcagtgcttcagccgctaccccgaccacatgaagcagcacgactt<br>cttcaagtccgccatgcccgaaggctacgtccaggagcgcaccatcttcttcaaggacgacggcaactacaa<br>gacccgcgccgaggtgaagttcgagggcgacaccctggtgaaccgcatcgagctgaagggcatcgacttca<br>aggaggacggcaacatcctggggcacaagCtggagtacaactacaacagccacaacgtctatatcatggc<br>cgacaagcagaagaacggcatcaaggtgaacttcaagatccgccacaacatcgaggacggcagcgtgca<br>gctcgccgaccactaccagcagaacacccccatcggcgacggccccgtgctgctgcccgacaaccactac<br>ctgagcacccagtccgccctgagcaaagaccccaacgagaagcgcgatcacatggtcctgctggagttcgt<br>gaccgccgccgggatcactctcggcatggacgagctgtacaagTGACTCTGATCCTGGCCAGA<br>AGGGAATGAGCGCCATGGGGTGGGGTGGGGTCATCAGGTA	This paper
dsDNA used as repair template for generation of GFP-*Tent5b* mouse line:<br>TTGGCCCGTGCACAGCCACTCTCCCTGCCCTCCTCGCCTTCACCATTCCC<br>GGGTTTCTGCCGTCCAGGCACCGGGGCCGGGTGAATGGTttCtAAGGGaGA<br>AGagctgttcacAggAgtggtgccTatcctggtcgagctggacggcgacgtaaacggccacaagttcagc<br>gtgtccggcgagggcgagggcgatgccacctacggcaagctgaccctgaagttcatctgcaccaccggcaa<br>gctgcccgtgccctggcccaccctcgtgaccaccctgacctacggcgtgcagtgcttcagccgctaccccgac<br>cacatgaagcagcacgacttcttcaagtccgccatgcccgaaggctacgtccaggagcgcaccatcttcttca<br>aggacgacggcaactacaagacccgcgccgaggtgaagttcgagggcgacaccctggtgaaccgcatcg<br>agctgaagggcatcgacttcaaggaggacggcaacatcctggggcacaagctggagtacaactacaacag<br>ccacaacgtctatatcatggccgacaagcagaagaacggcatcaaggtgaacttcaagatccgccacaaca<br>tcgaggacggcagcgtgcagctcgccgaccactaccagcagaacacccccatcggcgacggccccgtgct<br>gctgcccgacaaccactacctgagcacccagtccaagctgagcaaagaccccaacgagaagcgcgatca<br>catggtcctgctggagttcgtgaccgccgccgggatcactctcggcatggacgagctgtacaagggatCTgg<br>AGAAAACCTGTACTTCCAAGGAatgccATctgagagtggagctgaAagcctggagcagccag<br>ctgcgcaggtggggaccggtgcagcctcggcagtGGCCACGGCTG	This paper
dsDNA used as repair template for generation of *Tent5d*-FLAG mouse line:<br>CCACTGGTTTATTTCCAGCCATGTCATACAGTGCAGTTCCCTGTGCAAAATG<br>GTATGATGGACTACAAAGACGATGACGACAAGTAAGAAATACACATACCACA<br>AGTTTTGCTTAAGCAACTCTGAAAAAGCAATTTTCCAAGT	This paper
dsDNA used as repair template for generation of *Tent5d*-GFP mouse line:<br>CCACTGGTTTATTTCCAGCCATGTCATACAGTGCAGTTCCCTGTGCAAAATG<br>GTATGATGgagaatttgtattttcagggtgatatcatggtgagcaagggcgaggagctgttcaccggggtg<br>gtgcccatcctggtcgagctggacggcgacgtaaacggccacaagttcagcgtgtccggcgagggcgaggg<br>cgatgccacctacggcaagctgaccctgaagttcatctgcaccaccggcaagctgcccgtgccctggcccac<br>cctcgtgaccaccctgacctacggcgtgcagtgcttcagccgctaccccgaccacatgaagcagcacgacttc<br>ttcaagtccgccatgcccgaaggctacgtccaggagcgcaccatcttcttcaaggacgacggcaactacaag<br>acccgcgccgaggtgaagttcgagggcgacaccctggtgaaccgcatcgagctgaagggcatcgacttcaa<br>ggaggacggcaacatcctggggcacaagctggagtacaactacaacagccacaacgtctatatcatggccg<br>acaagcagaagaacggcatcaaggtgaacttcaagatccgccacaacatcgaggacggcagcgtgcagc<br>tcgccgaccactaccagcagaacacccccatcggcgacggccccgtgctgctgcccgacaaccactacctg<br>agcacccagtccgccctgagcaaagaccccaacgagaagcgcgatcacatggtcctgctggagttcgtgac<br>cgccgccgggatcactctcggcatggacgagctgtacaagTAAGAAATACACATACCACAAGT<br>TTTGCTTAAGCAACTCTGAAAAAGCAATTTTCCAAGT	This paper
Tn5ME-A:<br>CGTCGGCAGCGTCAGATGTGTATAAGAGACAG	Hennig et al.[64]
Tn5ME-B:<br>GTCTCGTGGGCTCGGAGATGTGTATAAGAGACAG	Hennig et al.[64]
Tn5MErev:<br>[phos]CTGTCTCTTATACACATCT	Hennig et al.[64]
Fw primer for SLIC cloning:<br>atgaacgagctctataagagatctttcgaaaaggacgagctgtaacaaagaaagcccagctccttc	This paper
Rv primer for SLIC cloning:<br>cacagtcgaggctgatcagcgggtttaaacaaagtgtgaaaaatacctctg	This paper

Fw oligonucleotide for Zp3 signal peptide sequence: agcttATGGCGTCAAGCTATTTCCTCTTCCTTTGTCTCCTGCTGTGTGGAGGCCCCGAGCTGTGCAATTCC	This paper
Rv oligonucleotide for Zp3 signal peptide sequence: CCGGGGAATTGCACAGCTCGGGGCCTCCACACAGCAGGAGACAAAGGAAGAGGAAATAGCTTGACGCCATa	This paper
Fw oligonucleotide for Gdf9 signal peptide sequence: agcttATGGCACTTCCCAGCAACTTCCTGTTGGGGGTTTGCTGCTTTGCCTGGCTGTGTTTTCTTAGTAGCCTTAGCTCTCAGGCTTCTACT	This paper
Rv oligonucleotide for Gdf9 signal peptide sequence: CCGGAGTAGAAGCCTGAGAGCTAAGGCTACTAAGAAAACACAGCCAGGCAAAGCAGCAAACCCCCAACAGGAAGTTGCTGGGAAGTGCCATa	This paper
Fw primer for plasmid linearization: tagagaacccactgcttactgg	This paper
Rv primer for plasmid linearization: TTTTTTTTTTTTTTTTTTTTggattgaaggagctgggcttt	This paper
Fw oligonucleotide for Zp3 PAT assay: GGGCCCCTGATATTCCTTGG	This paper
Fw oligonucleotide for Gdf9 PAT assay: CTGTGACCAGTCTCTCCGTG	This paper
Fw oligonucleotide for Tppp2 PAT assay: ACACCGGAACTCACAAGGAG	This paper
Fw oligonucleotide for Insl3 PAT assay: CGGTGCGTCCTTAATTGCTC	This paper
Rv universal oligonucleotide for PAT assay: CTTGCCTGTCGCTCTATCTT	This paper

Uncropped blots - Supplementary Figures

Sup. Figure 6. A - FLAG bands

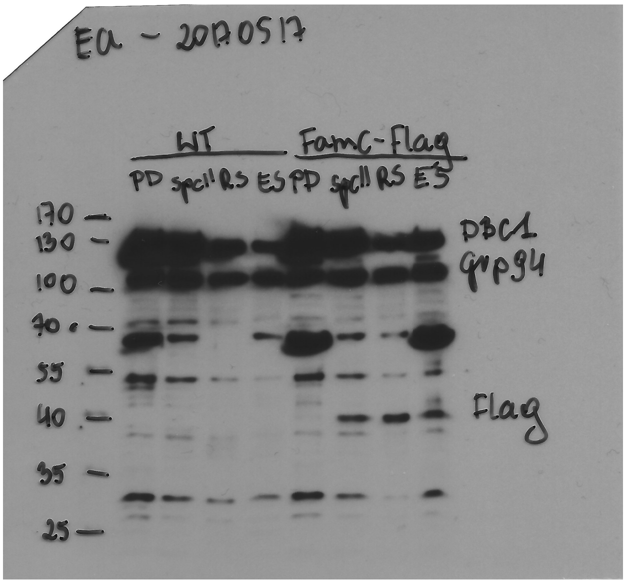

Sup. Figure 6. A - GRP-94 bands

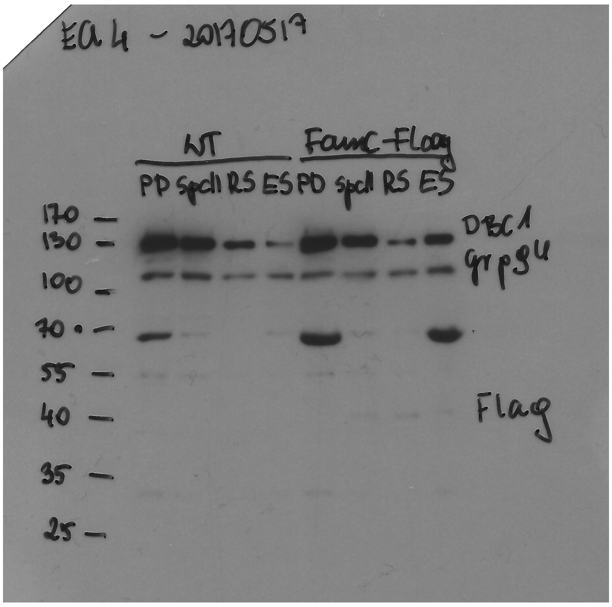

Sup. Figure 6. B - GRP-94 and FLAG bands

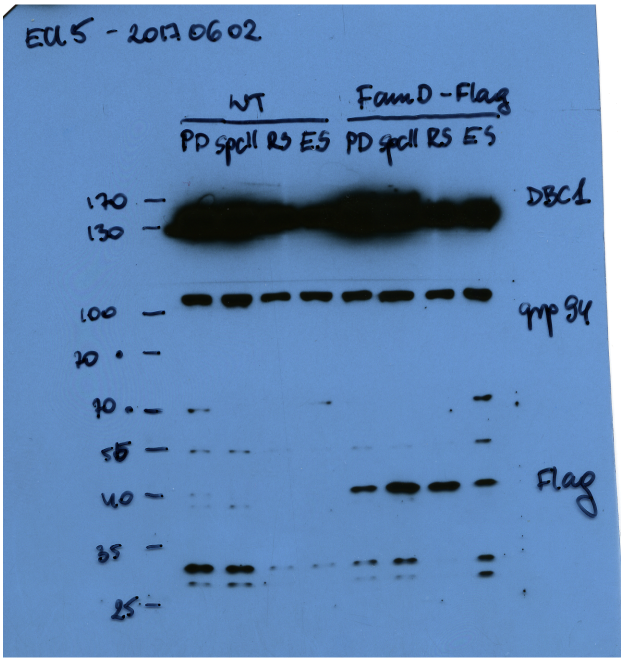

Sup. Figure 6. I

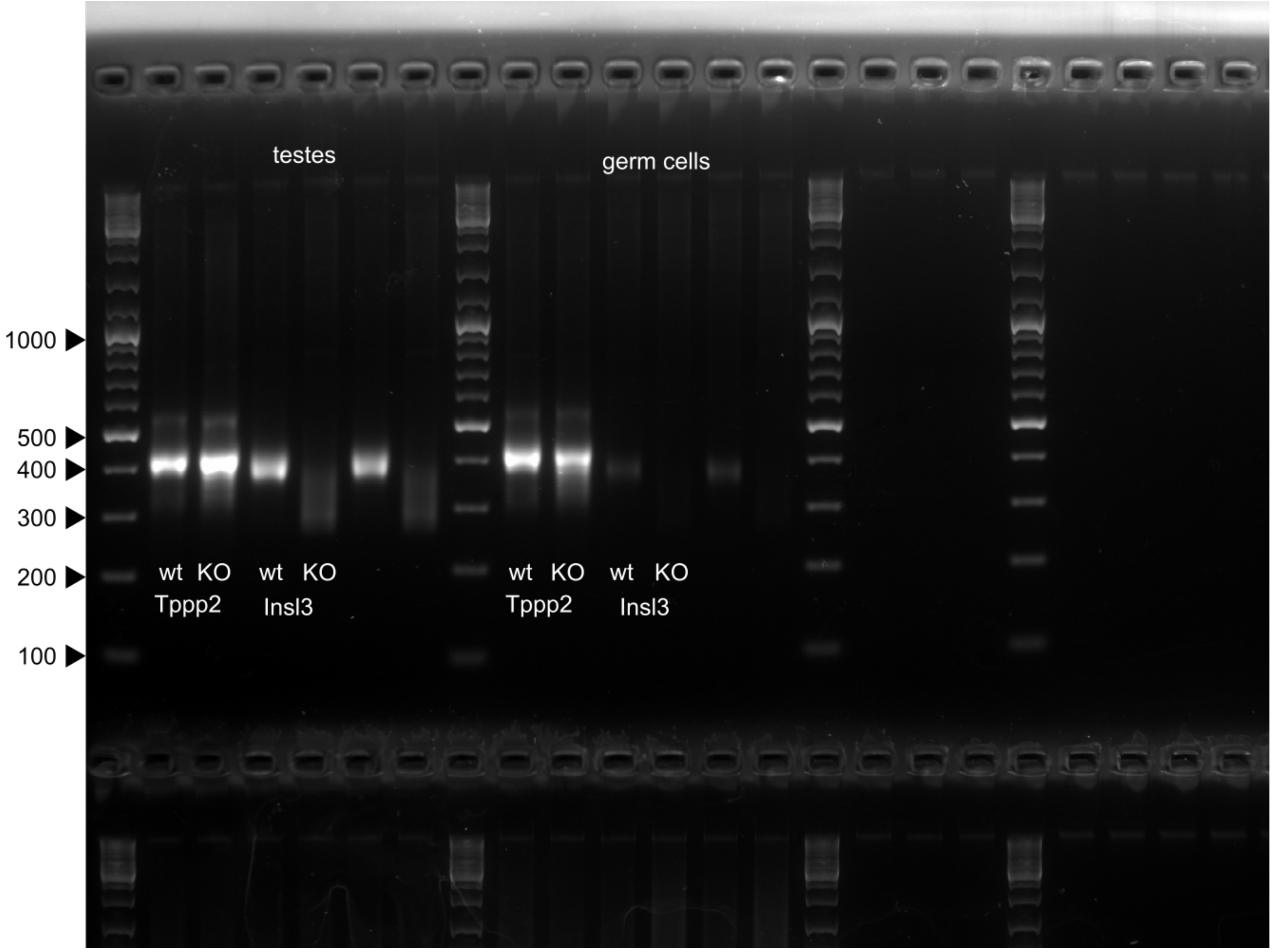

Supplement: Supplementary file 1 — Supplementary Information [file 41467_2024_49479_MOESM1_ESM.pdf]
