# Supplementary material for: TENT5-mediated polyadenylation of mRNAs encoding secreted proteins is essential for gametogenesis in mice
Source: Nat Commun. 2024 Jun 22;15:5331. doi: 10.1038/s41467-024-49479-4 (PMC11193744; doi:10.1038/s41467-024-49479-4)
Supplement: Supplementary file 3 — Description of Additional Supplementary Files [file 41467_2024_49479_MOESM3_ESM.pdf]

Description of Additional Supplementary Files

Supplementary Data Legends:

Supplementary Data 1: DRS run statistics and ENA accession numbers

Supplementary Data 2: Poly(A) tail length statisitics for RNA samples from Tent5b +/+ Tent5c +/+ (TENT5_WT) , Tent5b -/- Tent5c -/- (TENT5_BC_KO) ,Tent5b gfp/gfp (TENT5_GFP_hom) and Tent5b_+/gfp (TENT5_GFP_het) ovaries.

Supplementary Data 3: Results of differential polyadenylation analysis which includes potential targets of Tent5b and Tent5c. TENT5_BC_KO - Tent5b-/- Tent5c-/- , TENT5D_WT - Tent5b+/+ Tentc+/+. Significance of poly(A) tail difference were calculated using multiple pairwise comparisons in the Mann-Whitney-Wilcoxon test, two-sided. Padj were estimated using Benjamini-Hochberg method.

Supplementary Data 4: Results of differential polyadenylation analysis which includes potential targets of Tent5b. TENT5B_GFP_hom - Tent5b gfp/gfp , TENT5_WT - Tent5b wt/wt. Significance of poly(A) tail difference were calculated using multiple pairwise comparisons in the Mann-Whitney-Wilcoxon test, two-sided. Padj were estimated using Benjamini-Hochberg method.

Supplementary Data 5: Results of differential polyadenylation analysis on 522 oocyte specyfic genes. TENT5B_GFP_hom – Tent5b gfp/gfp, TENT5_WT – Tent5b +/+ Tent5c +/+ Significance of poly(A) tail difference were calculated using multiple pairwise comparisons in the Mann-Whitney-Wilcoxon test, two-sided. Padj were estimated using Benjamini-Hochberg method.

Supplementary Data 6: Results of differential polyadenylation analysis which includes potential targets of Tent5d. TENT5D_KO - Tent5d -/- , TENT5D_WT - Tent5d +/+ Significance of poly(A) tail difference were calculated using multiple pairwise comparisons in the Mann-Whitney-Wilcoxon test, two-sided. Padj were estimated using Benjamini-Hochberg method.

Supplementary Data 7: Results of differential polyadenylation analysis which includes potential targets of Tent5c. TENT5C_KO - Tent5c -/- , TENT5C_WT - Tent5c +/+ Significance of poly(A) tail difference were calculated using multiple pairwise comparisons in the Mann-Whitney-Wilcoxon test, two-sided. Padj were estimated using Benjamini-Hochberg method.
