# Supplementary material for: TENT5-mediated polyadenylation of mRNAs encoding secreted proteins is essential for gametogenesis in mice
Source: Nat Commun. 2024 Jun 22;15:5331. doi: 10.1038/s41467-024-49479-4 (PMC11193744; doi:10.1038/s41467-024-49479-4)
Supplement: Supplementary file 11 — Reporting Summary [file 41467_2024_49479_MOESM11_ESM.pdf]

nature portfolio

Corresponding author(s): Andrzej Dziembowski

Last updated by author(s): Mar 6, 2024

Reporting Summary

Nature Portfolio wishes to improve the reproducibility of the work that we publish. This form provides structure for consistency and transparency in reporting. For further information on Nature Portfolio policies, see our Editorial Policies and the Editorial Policy Checklist.

Statistics

For all statistical analyses, confirm that the following items are present in the figure legend, table legend, main text, or Methods section.

n/a	Confirmed	
☐	☒	The exact sample size (*n*) for each experimental group/condition, given as a discrete number and unit of measurement
☐	☒	A statement on whether measurements were taken from distinct samples or whether the same sample was measured repeatedly
☐	☒	The statistical test(s) used AND whether they are one- or two-sided<br>*Only common tests should be described solely by name; describe more complex techniques in the Methods section.*
☐	☒	A description of all covariates tested
☐	☒	A description of any assumptions or corrections, such as tests of normality and adjustment for multiple comparisons
☐	☒	A full description of the statistical parameters including central tendency (e.g. means) or other basic estimates (e.g. regression coefficient) AND variation (e.g. standard deviation) or associated estimates of uncertainty (e.g. confidence intervals)
☐	☒	For null hypothesis testing, the test statistic (e.g. *F*, *t*, *r*) with confidence intervals, effect sizes, degrees of freedom and *P* value noted<br>*Give P values as exact values whenever suitable.*
☒	☐	For Bayesian analysis, information on the choice of priors and Markov chain Monte Carlo settings
☒	☐	For hierarchical and complex designs, identification of the appropriate level for tests and full reporting of outcomes
☒	☐	Estimates of effect sizes (e.g. Cohen's *d*, Pearson's *r*), indicating how they were calculated

Our web collection on statistics for biologists contains articles on many of the points above.

Software and code

Policy information about availability of computer code

Data collection	FACS Diva Software v8.0.1 (BD) was used to collect data during cell sorting. MinKNOW 19.10.1 (MinKNOW core 3.5.5; Bream 4.2.11; GUI 3.5.10) was used for data colection during ONT RNA sequencing. ZEN 3.6 software was used for microscopy image aquisition. Harmony High-Content Imaging and Analysis Software (PerkinElmer) was used for oocyte screening.
Data analysis	FlowJo (Data Analysis Software v10; BD); Fiji (Is Just ImageJ) [ImageJ ver. 1.53-1.54]; R Project for Statistical Computing (v. 3.6.0); RStudio (ver. 2023.12.1+402); Minimap2 (2 2.17); samtools (1.9); Nanopolish (0.13.2); featureCounts; STAR; DESeq2 (3.17); bedtools (2.29.2); UCSC Table Browser tool; STREME; biomaRt R library; TargetP; seqtk; R ggplot library;

For manuscripts utilizing custom algorithms or software that are central to the research but not yet described in published literature, software must be made available to editors and reviewers. We strongly encourage code deposition in a community repository (e.g. GitHub). See the Nature Portfolio guidelines for submitting code & software for further information.

Data

Policy information about availability of data

All manuscripts must include a data availability statement. This statement should provide the following information, where applicable:

- Accession codes, unique identifiers, or web links for publicly available datasets
- A description of any restrictions on data availability
- For clinical datasets or third party data, please ensure that the statement adheres to our policy

Plasmids, mouse lines and reagents generated in this study are available at request from corresponding author.
Supplementary Figures 1 to 9 together with Supplementary Table 1 containing oligonucleotides, dsDNA, and sgRNA sequences, are available in Supplementary Information file.
Results of sequencing data analyses are available as Supplementary Data 1-7.
Source data are provided with this paper, containing data used for plot generation (except RNA-seq data) and uncropped pictures of blots and gels.
All DRS sequencing data generated in this study have been deposited in the European Nucleotide Archive (ENA) database under accession code PRJEB63526 (https://www.ebi.ac.uk/ena/browser/view/PRJEB63526).
The raw Illumina RNAseq data have been deposited in Gene Expression Omnibus (GEO) database under accession code GSE239661 (https://www.ncbi.nlm.nih.gov/geo/query/acc.cgi?acc=GSE239661).
Additionally, ENA sample accession numbers together with DRS run details are listed in Supplementary Data 1.
Any additional information required to reanalyse the data reported in this paper is available from the corresponding author upon request.

Research involving human participants, their data, or biological material

Policy information about studies with human participants or human data. See also policy information about sex, gender (identity/presentation), and sexual orientation and race, ethnicity and racism.

Reporting on sex and gender	Not applicable
Reporting on race, ethnicity, or other socially relevant groupings	Not applicable
Population characteristics	Not applicable
Recruitment	Not applicable
Ethics oversight	Not applicable

Note that full information on the approval of the study protocol must also be provided in the manuscript.

Field-specific reporting

Please select the one below that is the best fit for your research. If you are not sure, read the appropriate sections before making your selection.

☒ Life sciences ☐ Behavioural & social sciences ☐ Ecological, evolutionary & environmental sciences

For a reference copy of the document with all sections, see nature.com/documents/nr-reporting-summary-flat.pdf

Life sciences study design

All studies must disclose on these points even when the disclosure is negative.

Sample size	The sample size is provided in Source Data files and figures. No statistical methods were used to predetermine sample size. For in vivo animal experiments, we estimated the sample size based on previous studies, our pilot experiments.
Data exclusions	There was no formal criteria of sample exlusions. However, samples with clear technical failures during tissue harvesing, cells isolation, processing or data collection throught flow cytometry or biochemical experiments were excluded from analyses.
Replication	For all quantitative experiments data are representative of multiple independent experiments. For RNA-seq we used 3 replicates, which is the current standard in the literature. For other experiments we refered to literature data. Most of the experiments were repeated at least twice and material from at least two animals was used, leading to comparable results with an exception of of Tent5d KO germ cell distribution measurements across mice life, where only one male was used per life timepoint.The exact number of biological replicates used for statistical analysis is stated for every single experiment. As described above, samples with clear technical failures during processing or data collection were excluded from analyses.
Randomization	Mice of different genotypes were assigned with individual numerical tags in the database and they were used for the tissue collection and throughout subsequent processing as the only identifiers.
Blinding	Individual mice had an assigned numerical tag and mice with different genotypes were indistinguishable by the experimenter.

Reporting for specific materials, systems and methods

We require information from authors about some types of materials, experimental systems and methods used in many studies. Here, indicate whether each material, system or method listed is relevant to your study. If you are not sure if a list item applies to your research, read the appropriate section before selecting a response.

Materials & experimental systems

n/a	Involved in the study
☐	☒ Antibodies
☒	☐ Eukaryotic cell lines
☒	☐ Palaeontology and archaeology
☐	☒ Animals and other organisms
☒	☐ Clinical data
☒	☐ Dual use research of concern
☒	☐ Plants

Methods

n/a	Involved in the study
☒	☐ ChIP-seq
☐	☒ Flow cytometry
☒	☐ MRI-based neuroimaging

Antibodies

Antibodies used

Monoclonal Anti-β-Tubulin - Sigma-Aldritch - Cat# F2043 Lot: 108M4766V
INSL3 Polyclonal Antibody - Thermo Fisher Scientific - Cat# PA5-55921 Lot: A96525
Anti-RNASET2 antibody produced in rabbit - Sigma-Aldritch - Cat# HPA029013 Lot: A115851
Anti-TPPP2 antibody - Abcam - Cat# ab236887
Recombinant Anti-GDF 9 antibody - Abcam - Cat# ab254323 Lot: GR3279299-2
ZP3 Polyclonal antibody - Proteintech - Cat# 21279-1-AP Lot: 00041356
Donkey anti-Rabbit IgG (H+L) Highly Cross-Adsorbed Secondary Antibody, Alexa Fluor™ 568 - Thermo Fisher Scientific - Cat# A-10042 Lot: 2136776
Goat anti-Rabbit IgG (H+L) Cross-Adsorbed Secondary Antibody, Alexa Fluor™ 568 - Thermo Fisher Scientific - Cat# A-11011 Lot: 2273773
DYKDDDDK Tag Polyclonal Antibody - Thermo Fisher Scientific - Cat# PA1-984B Lot: WG319616
GRP 94 Antibody (H-212) - Santa Cruz Biotechnology - Cat# sc-11402 Lot: C11616
Goat Anti-Rabbit IgG, H & L Chain Specific Peroxidase Conjugate - Millipore - Cat# 401393 Lot: 3924034

Validation

Monoclonal Anti-β-Tubulin - this antibody was used by us in multiple previous projects, and proved to work without any unspecific stainings. Additionaly β-Tubulin staining is very characteristic, making initial observation of staining results sufficient to determine it's validity. No validation information is available on manufacturers website.

DYKDDDDK Tag Polyclonal Antibody - antibody performance assesment was performed by using biological material from wild-type mice without FLAG-tag as a negative control

GRP 94 Antibody (H-212) - antibody tested extensively by us within different mouse tissues as a western-blot loading control, manufacturer provides number of scientific publications citing usage of this antibody in western-blot technique (https://www.scbt.com/p/grp-94-antibody-h-212)

Recombinant Anti-GDF 9 antibody - manufacturer provides performance validation in immuhistochemistry in mice ovarian samples (https://www.abcam.com/en-pl/products/primary-antibodies/gdf-9-antibody-epr22495-129-ab254323). Our staining was clear and oocyte specific, devoided on any unspecific staining in other cells

ZP3 Polyclonal antibody - manufacturer provides performance validation in immunohistochemistry in mice ovarian samples (https://www.ptglab.com/products/ZP3-Antibody-21279-1-AP.htm). Our staining was clear and oocyte specific. Stained Zona Pelucida 3 protein also displays very characteristic localization in the oocyte, which is visible in our staining.

INSL3 Polyclonal Antibody - manufacturer provides validation in immunihistochemistry in mice testis tissues samples (https://www.thermofisher.com/antibody/product/INSL3-Antibody-Polyclonal/PA5-55921). Our staining recapitulates staining pattern provided by this validation.

Anti-RNASET2 antibody produced in rabbit - manufacturer provides extensive validation of specificity of this antibody towards desired target.(https://www.sigmaaldrich.com/PL/pl/product/sigma/hpa029013). No data for mice testis tissues samples is provided.

Anti-TPPP2 antibody - manufacturer provides performance validation in immunohistochemistry in mice samples (https://www.abcam.com/en-pl/products/primary-antibodies/tppp2-antibody-ab236887). No data for mice testis tissues samples is provided.

Animals and other research organisms

Policy information about studies involving animals; ARRIVE guidelines recommended for reporting animal research, and Sex and Gender in Research

Laboratory animals

All experiments were performed using mouse lines generated using CRISPR/Cas9 method in C57BL/6/Tar x CBA/Tar mixed

Laboratory animals	background.<br>In this study females of following genotype and age were used: Tent5a-/- (8-12 weeks), Tent5b-/-c-/- (4-12 weeks), Tent5b-/-c+/- (8-12 weeks), Tent5bgfp/gfp (C-terminal) (8-12 weeks), Tent5bgfp/gfp (N-terminal) (4-12 weeks), Tent5bgfp/wt (N-terminal) (4-12 weeks), Tent5cgfp/gfp (8-12 weeks), wild-type (4-12 weeks), and males of following genotype and age were used: Tent5bgfp/gfp (8-48 weeks), Tent5c-/- (8-48 weeks), Tent5dnull/- (3-48 weeks), Tent5cgfp/gfp (8-48 weeks), Tent5cflag/flag (8-48 weeks), Tent5dgfp/gfp (3 weeks), Tent5dflag/flag (8-48 weeks), wild-type (3-48 weeks).
Wild animals	This study did not involve wild animals
Reporting on sex	Sex of animals studied was solely determined by fertility phenotype observed.
Field-collected samples	Study did not involve samples collected from the field.
Ethics oversight	All animal experiments were approved by the Local Ethical Committees in Warsaw affiliated to the University of Warsaw, Faculty of Biology (approval numbers: 176/2026, 917/2019) and Warsaw University of Life Sciences, Faculty of Horticulture and Biotechnology (approval numbers: WAW2/049/2022) and were performed according to Polish Law (Act number 266/15.01.2015).

Note that full information on the approval of the study protocol must also be provided in the manuscript.

Plants

Seed stocks	Not applicable
Novel plant genotypes	Not applicable
Authentication	Not applicable

Flow Cytometry

Plots

Confirm that:

☒ The axis labels state the marker and fluorochrome used (e.g. CD4-FITC).

☒ The axis scales are clearly visible. Include numbers along axes only for bottom left plot of group (a 'group' is an analysis of identical markers).

☒ All plots are contour plots with outliers or pseudocolor plots.

☒ A numerical value for number of cells or percentage (with statistics) is provided.

Methodology

Sample preparation	For male germ cells isolation from testis, a protocol adapted from the work of Bastos et al. was used. Dissected testes were decapsulated and placed in Falcon with 25 ml of HSBB buffer (Gibco) at room temperature. Next, 1 ml of type XI collagenase (12.5 mg/ml) was added (Sigma) and tubes were put to shake at 32°C for 20 minutes in the shaking water bath (120 osc/min). Tubes were manually agitated every 5 minutes to facilitate the dissociation of the tubules. The tubules were washed once in 25 ml 1x HSBB buffer and resuspended in 10 ml of the HSBB buffer with 200 µl of the stock trypsin (25 mg/ml, Sigma) and 2 µl of the stock DNAse I (5 mg/ml, Sigma) and again put in a water bath with agitation at 32°C for 15 minutes (120 osc/min). After the incubation, samples were dissociated for 4 minutes by pipetting 10 ml serological pipet and centrifuged at 1500 rpm for 3 min. The supernatant was discarded, and cells were resuspended in 1 ml of HSBB with 10% FBS (Gibco) and counted in Thoma chamber. Prepared cells were used for further procedures.<br>For cell cycle analysis germ cells were stained with Vybrant™ DyeCycle™ Violet Stain (Thermo Fisher Scientific, Cat# V35003) 1 µl per 1 mln of germ cells62. Cells were incubated at 32°C in a water bath with agitation for 35 minutes (osc 90/min). Fluorescence was excited by 405 nm laser and DCV Blue fluorescence was detected with 450/50 filters while DCV Red fluorescence was detected with 525/50 filters. For the live/dead staining, the LIVE/DEAD™ Fixable Near-IR Dead Cell Stain Kit (Thermo Fisher Scientific, Cat# L34976) was used. Fluorescence of GFP was excited by 488 nm laser and detected with 530/30 filters.
Instrument	Samples were analyzed with BD LSRFortessa™ and sorted with BD Aria Fusion™
Software	FACS Diva Software v8.0.1 (BD) software was used during sample acquireing and samples were analyzed using FlowJo (Data Analysis Software v10).
Cell population abundance	500 000 - 1000 000 cells were collected for each sample

Gating strategy

Gating strategy was as follow: cells -> singlets fsc -> singlest ssc -> live cells -> different population of male sperm cells were gated (gonia, 4C, spcII, RS and ES) visualised in Supplementary Figure 5.

☒ Tick this box to confirm that a figure exemplifying the gating strategy is provided in the Supplementary Information.
